# Current News

**Published:** 1890-02

**Authors:** 


### Current News.

We are informed that a *Dental Anatomy* by a well known member of the profession is now in process of preparation which, when completed, will furnish a much-needed text-book upon that subject, and, coming from the source it does, will be a standard work.

---

Massachusetts State Dental Society.—The following officers were elected in the Massachusetts State Dental Society at its twenty-fifth annual meeting, held in Boston, December 12 and 13, 1889. President, Robert R. Andrews, D.D.S, Cambridge, Mass.; First Vice-President, Geo. F. Eames, M.D., D.D.S., Boston; Second Vice-President, J. W. Ball, D.D.S.; Secretary, Edgar O. Kinsman, D.D.S., Cambridge, Mass.; Treasurer, Edward Page, M.D., D.M.D., Charlestown, Mass.; Librarian, Jos. King Knight, D.D.S., Boston.

*Executive Committee.*—Drs. D. M. Clapp, Boston; G. A. Gerry, Lowell; E. W. Branigan, Boston; W. E. Boardman, Boston; H. S. Draper, Boston.

---

The Fiftieth Commencement of the Baltimore College of Dental Surgery will be held on Thursday, March 20, 1890. All graduates and friends of the College are invited to be present.

---

The new Dental Crown of Dr. W. H. Gates, which is just now attracting marked attention, will appear as the culmination of the subject of "Crown Mounting" in the forthcoming new edition of Garretson's Oral Surgery.

---

Dr. Atkinson says the expression of *physical pain* has no meaning. We know pain only by the uncomfortable movement of what we call consciousness, and so, of all that we know, it is simply a mode of consciousness. If the word *physical* could be wiped out, it would do away with much of our misapprehension.

---

Dr. A. H. Thompson says that his cure for sensitive dentine is a temporary stopping of oxyphosphate for a week or two. He has found it reliable and satisfactory for all cavities in sensitive dentine.

